# Clinical and Survival Impact of Sex-Determining Region Y-Box 2 in Colorectal Cancer: An Integrated Analysis of the Immunohistochemical Study and Bioinformatics Analysis

**DOI:** 10.1155/2020/3761535

**Published:** 2020-02-13

**Authors:** Kun Song, Jingduo Hao, Zuyin Ge, Pushi Chen

**Affiliations:** ^1^Department of General Surgery, People's Hospital of Zhenhai, Ningbo, Zhejiang 315202, China; ^2^Health Management Center, Ningbo First Hospital, Ningbo, Zhejiang 315000, China

## Abstract

Transcription factor sex-determining region Y-box 2 (SOX2) involves in the maintenance of cancer stem cells. However, the role of SOX2 in colorectal cancer (CRC) remains unclear. This study was conducted to investigate the effect of SOX2 on CRC. Studies were searched using electronic databases. The combined odds ratios (ORs) or hazard ratios (HRs: multivariate Cox survival analysis) with their 95% confidence intervals (CIs) were calculated. The Cancer Genome Atlas (TCGA) and GEO datasets were further applied to validate the survival effect. The functional analysis of SOX2 was investigated. In this work, 13 studies including 2337 patients were identified, and validation data were enrolled from TCGA and GEO datasets. SOX2 expression was not significantly related to age, gender, microsatellite instability (MSI) status, clinical stage, histological grade, tumor size, pT-stage, lymph node metastasis, distal metastasis, and cancer-specific survival (CSS) but was correlated with worse overall survival (OS: *n* = 536 patients) (*P* < 0.05). Furthermore, TCGA data demonstrated similar results, with no significant correlation between SOX2 and pathological characteristics. Further validation data (OS: *n* = 1408 and disease-free survival (DFS): *n* = 1367) showed that SOX2 expression was correlated with worse OS (HR = 1.35, 95% CI: 1.11–1.65, *P*=0.004) and DFS (HR = 1.30, 95% CI: 1.04–1.62, *P*=0.02). The functional analyses showed that SOX2 involved in cell-cell junction, focal adhesion, extracellular matrix- (ECM-) receptor interaction, and MAP kinase activity. Our findings suggest that SOX2 expression may be correlated with the worse prognosis of CRC.

## 1. Introduction

Colorectal cancer (CRC) is one of the most common malignant tumors and a major cause of cancer-related death in the world and in China [1, 2]. According to the GLOBOCAN estimates, approximately 1,800,977 new cases were diagnosed with CRC, leading to approximately 861,663 deaths due to this disease in 2018 worldwide [1]. Although some significant achievements have been made in early detection and treatment in recent years, most patients are diagnosed with advanced disease and show a poor 5-year survival rate [3–5]. Therefore, it is needed to find new efficient biomarkers for the treatment of CRC along with the prognosis.

Cancer stem cells (CSCs) are a special subpopulation of tumor cells and have the ability and characteristics of self-renewal and multilineage differentiation and proliferation potential, which are related to tumor progression, metastasis, recurrence, prognosis, and drug resistance [6–8]. Many CSC-related markers have been identified and reported to be associated with poor prognosis and resistance to therapy in cancer [9, 10]. Sex-determining region Y-box 2 (SOX2), a high mobility group (HMG) DNA-binding domain, is mapped to human chromosome 3q26.3–q27 and belongs to a key transcription factor [11]. SOX2 regulates the self-renewal and pluripotency of undifferentiated stem cells such as human embryonic stem cells and plays an important role in maintaining the stem cell–like features in cancer cells [12–14]. Additionally, SOX2 involves in the migration, invasion, and proliferation of cancer cells and resistance to therapy [15, 16]. SOX2 has been reported to be commonly expressed in many human cancers, such as breast cancer, lung cancer, esophageal cancer, and CRC [17]. For example, SOX2 expression is correlated with worse prognosis in breast cancer and gastric cancer [18, 19]. While SOX2 expression shows a favorable prognosis in non-small cell lung cancer and cervical cancer [20, 21]. Therefore, it is of great importance to determine the prognostic role of SOX2 expression in CRC.

The previous meta-analysis only included eight studies with small sample sizes (*n* = 1113) and only analyzed the correlation between SOX2 and overall survival (OS) and a small part of the clinical features of CRC. Additionally, the result on OS was not reasonable [22]. Therefore, the significance of SOX2 expression in CRC is not fully understood. For example, Lundberg et al. reported that SOX2 expression was not related to cancer-specific survival in CRC [23], but SOX2 expression was correlated with worse cancer-specific survival by Miller et al. [24]. Thus, the current work with 3745 CRCs was performed to determine the survival impact of SOX2 expression on CRC ((cancer-specific survival (CSS), overall survival (OS), and disease-free survival (DFS)) and to evaluate the association between SOX2 expression and general clinicopathological characteristics.

## 2. Materials and Methods

### 2.1. Literature Search

This meta-analysis was performed based on the Preferred Reporting Items for Systematic reviews and Meta-Analyses (PRISMA) statement [25] (Supplementary Materials). The electronic databases PubMed, EMBASE, and Web of Science were comprehensively searched to identify eligible publications until July 29, 2019, by searching the following key words and search terms: “colorectal cancer OR colorectal tumor OR colorectal carcinoma OR colorectal neoplasm OR CRC OR rectal cancer OR rectal tumor OR rectal carcinoma OR colon cancer OR colon tumor OR colon carcinoma” and “SOX2 OR SOX-2 OR Sex-determining region Y-box protein 2 OR Sex determining region Y box-2 OR Sex-determining region Y-box 2 OR SRY box-2” (Supplementary Materials). Moreover, the reference lists of the identified articles were also examined to find other relevant studies.

### 2.2. Eligibility Criteria

For the enrollment of publications, the main inclusion criteria were included as follows: (1) the patients with CRC were reported; (2) studies reported the detection of SOX2 using immunohistochemistry (IHC); (3) expression status of SOX2 was defined from the original articles; (4) studies provided available data to assess the correlation between SOX2 expression and clinicopathological parameters; (5) studies provided sufficient hazard ratio (HR) with 95% confidence interval (CI) to evaluate the prognostic impact of SOX2 expression on CRC patients based on multivariate Cox survival analysis. If the results of interest were not completely reported, the corresponding author will be contacted via e-mail. Only the recent article or the article with the most complete data was selected when multiple articles using overlapping tissue samples from the same institute were published. We mainly excluded review articles, letters, conference abstracts, case reports, cell line/animal studies, and articles lacking sufficient data.

### 2.3. Data Extraction and Study Quality Assessment

The relevant data and search of the included studies were conducted from two independent authors (KS and JH) using standardized forms, including the surname of the first author, time of publication, country, ethnicity, median/mean age, disease stage, antibody information, number of participants, cutoff values of SOX2, expression frequency, clinicopathological parameters such as age, gender, microsatellite instability (MSI) status, clinical stage, histological grade, tumor size, pT-stage, lymph node metastasis, and distal metastasis, and the survival data of multivariate Cox analysis such as cancer-specific survival (CSS), overall survival (OS), and disease-free survival (DFS). The quality of the available publications was assessed according to the Newcastle–Ottawa Scale (NOS) for cohort studies [26, 27]. Three parameters of quality included a total of nine scores: selection (0–4), comparability (0–2), and outcome assessment (0–3). In this meta-analysis, the publication with ≥6 scores was considered to be of high quality, and the NOS score with <6 was defined as low-quality study. Any disagreements in the selected literature were discussed by all authors and then reached a consensus.

### 2.4. Validation Data from TCGA

The RNA-sequencing data and corresponding clinical information on CRC were obtained from The Cancer Genome Atlas (TCGA) (https://portal.gdc.cancer.gov/repository). Eventually, 618 cases with CRC with sufficient expression data and clinical information were selected.

### 2.5. Functional Analysis of SOX2

Association between SOX2 and genes was analyzed using TCGA sequencing data. Spearman coefficients with an absolute value of >0.2 and *P* < 0.001 were applied for SOX2. The potential function and biological mechanism of the SOX2 gene such as GO (Gene Ontology) and KEGG (Kyoto Encyclopedia of Genes and Genomes) pathways were investigated by clusterProfiler package.

### 2.6. Survival Analysis from Validation Datasets

Normalized GSE17538 (*n* = 232 patients), GSE39582 (*n* = 558 patients), and TCGA (*n* = 618 patients) datasets were applied due to sufficient survival information and the batch effects were adjusted using the ComBat method [28, 29]. Finally, 1408 CRC patients were used to further confirm whether SOX2 expression was still correlated with worse OS and 1367 CRC patients were used to further validate whether SOX2 expression was still related to disease-free survival (DFS). The optimal cutoff value (SOX2: 3.31) was selected and survival analysis was performed using “survival and survminer” packages.

### 2.7. Statistical Analysis

Data on meta-analysis were obtained from the original articles. The pooled odds ratios (ORs) and the corresponding 95% CIs were calculated to assess the relationship of SOX2 expression with the clinicopathological parameters. The pooled HRs with their 95% CIs were performed to estimate the prognostic impact of SOX2 expression on CRC patients using multivariate Cox survival analysis. As described in the report of IntHout et al., the Hartung–Knapp–Sidik–Jonkman (HKSJ) method was applied to improve the reliability of the pooled results with ≤10 studies in the current meta-analysis [30]. The heterogeneity assumption between studies was measured using Cochran's *Q* statistic [31]. A *Q* test of *P* < 0.1 was considered to be a significant heterogeneity. When substantial heterogeneity was detected, sensitivity analyses were conducted to estimate the change of heterogeneity and stability of an individual study on the recalculated results by removing a single study [32]. The possible publication bias was performed by Egger's test [33]. The pooled ORs and HRs were calculated using “metafor” package via R version 3.5.1 [R Core Team, 2018]. Results of publication bias were carried out using Stata software (version 12.0, Stata Corporation, College Station, TX, US). The code for the relevant examples is listed in Supplementary Materials.

For bioinformatics validation data, the TCGA patients were divided into positive and negative groups based on the median value of SOX2 expression. The relationships between SOX2 expression and the clinicopathological parameters were performed using the univariate logistic regression analysis. The clinicopathological parameters included age, MSI status, clinical stage, lymph node metastasis, distal metastasis, venous invasion, and lymphatic invasion. Analysis for validation data was performed by using R version 3.5.1 [R Core Team, 2018].

## 3. Results

### 3.1. Study Characteristics

**Figure 1:**
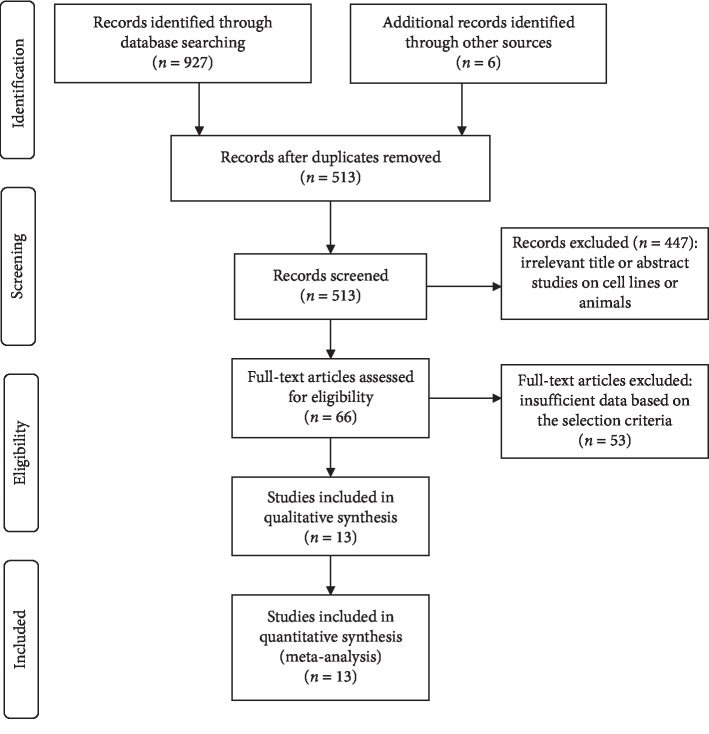
Flow diagram of the study selection process.

The flow diagram for the study selection is presented in Figure 1. Based on the selection criteria, inappropriate studies were excluded. Finally, 13 studies published between 2010 and 2019 were identified in the present meta-analysis [6, 14, 23, 24, 34–42], including 2337 patients with CRC. Eligible studies were conducted in China, Korea, Singapore, Australia, Germany, Sweden, and The Netherlands. Among these studies, expression of SOX2 was evaluated by IHC. 10 studies with 1431 cases evaluated the relationship between SOX2 expression and the clinicopathological parameters [14, 23, 24, 35–41]. Five studies with 1451 cases assessed the association between SOX2 expression and the prognosis using multivariate Cox survival analysis [6, 23, 24, 34, 42]. 13 studies were considered to be of high quality by using NOS. The details of the included studies are summarized in Table 1 and [Supplementary-material supplementary-material-1].

### 3.2. Correlation of SOX2 Expression with Clinicopathological Variables

**Figure 2:**
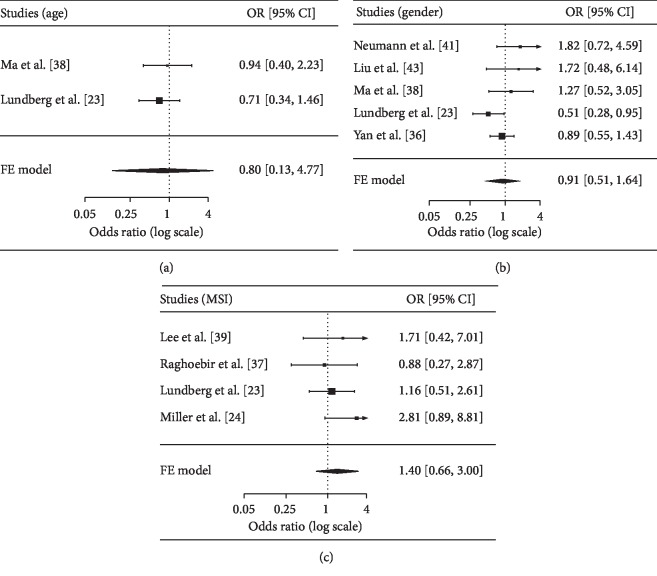
Forest plot for the correlation between SOX2 and age, gender, and MSI status. (a) Age (≥60 vs. <60 years); (b) gender (male vs. female); (c) MSI status (MSI vs. MSS). OR: odds ratio; CI: confidence interval; MSS: microsatellite stability; MSI: microsatellite instability.

A summary of the calculated results is shown in Table 2. No association was found between SOX2 expression and age (two studies with 530 cases, ≥60 vs. <60 years: OR = 0.80, *P*=0.352), gender (five studies with 991 cases, male vs. female: OR = 0.91, *P*=0.684), MSI status (four studies with 775 cases, MSI vs. microsatellite stability (MSS): OR = 1.40, *P*=0.25) (Figure 2).

**Figure 3:**
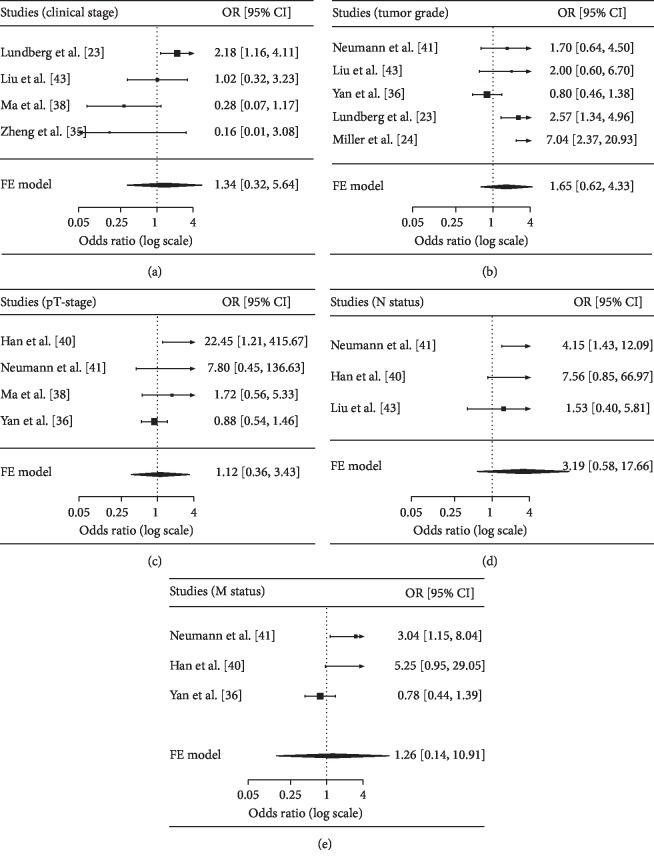
Forest plot for the correlation between SOX2 and clinical stage, histological grade, pT-stage, lymph node metastasis, and distal metastasis. (a) Clinical stage: stages 3-4 vs. 1-2; (b) tumor grade: grades 3-4 vs. 1-2; (c) pT-stage: T 3-4 vs. 1-2; (d) lymph node metastasis (N status): yes vs. no; (e) distal metastasis (M status): yes vs. no. OR: odds ratio; CI: confidence interval.

No correlation was found between SOX2 expression and clinical stage (four studies with 619 cases, stages 3-4 vs. 1-2: OR = 1.34, *P*=0.564) and histological grade (five studies with 999 cases, grades 3-4 vs. 1-2: OR = 1.65, *P*=0.227) (Figure 3).

No relationship was observed between SOX2 expression and tumor size (two studies with 347 cases, ≥5 vs. ≤5 cm: OR = 0.78, *P*=0.759), pT-stage (four studies with 526 cases, pT-stages 3-4 vs. 1-2: OR = 1.12, *P*=0.778), lymph node metastasis (three studies with 225 cases, yes vs. no: OR = 3.19, *P*=0.1), and distal metastasis (three studies with 438 cases, yes vs. no: OR = 1.26, *P*=0.694) (Figure 3).

### 3.3. Survival Impact of SOX2 Expression Using Multivariate Cox Analysis

**Figure 4:**
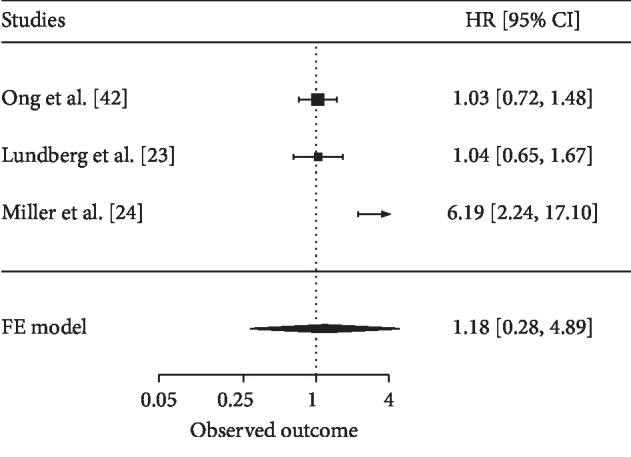
Forest plot for the correlation between SOX2 and cancer patients' prognosis using multivariate cox analysis in cancer-specific survival (CSS). HR: hazard ratio; CI: confidence interval.

Data showed that SOX2 expression was not correlated with cancer-specific survival (CSS) of CRC in three studies with 855 patients (HR = 1.18, *P*=0.667) (Figure 4) but was correlated with worse overall survival (OS) in two studies with 536 patients (*P* < 0.05) [24, 34].

### 3.4. Heterogeneity Analysis

Heterogeneity analysis was conducted between SOX2 expression and clinical stage, histological grade, and pT-stage. We conducted sensitivity analyses to estimate the change of the pooled results. When the study of Lundberg et al. [23] was deleted, the recalculated OR was still not correlated with the clinical stage (OR = 0.55, 95% CI = 0.06–4.66, *P*=0.350), with no evidence of heterogeneity (*P*=0.272). When the study of Han et al. [40] was omitted, the recalculated result was still not associated with the pT-stage (OR = 1.04, 95% CI = 0.30–3.54, *P*=0.908), with no heterogeneity (*P*=0.216). When the study of Yan et al. [36] was removed, the recalculated result was significantly correlated with the histological grade (OR = 2.70, 95% CI = 1.15–6.37, *P*=0.035), with no heterogeneity (*P*=0.250).

### 3.5. Publication Bias

No publication bias was found between SOX2 expression and gender and histological grade (*P* > 0.1) ([Supplementary-material supplementary-material-1]).

### 3.6. TCGA

Six hundred eighteen cases with CRC were enrolled from TCGA. As shown in Table 3, the results using univariate logistic regression analysis showed that SOX2 expression was not significantly associated with age (≥60 vs.<60 years: OR = 1.03, 95% CI = 0.73–1.46, *P*=0.859), MSI status (MSI vs. MSS : OR = 1.08, 95% CI = 0.77–1.52, *P*=0.663), clinical stage (stages 3-4 vs. 1-2: OR = 1.37, 95% CI = 0.99–1.89, *P*=0.059), venous invasion (yes vs. no: OR = 1.12, 95% CI = 0.75–1.65, *P*=0.585), lymphatic invasion (yes vs. no: OR = 1.27, 95% CI = 0.91–1.79, *P*=0.162), lymph node metastasis (yes vs. no: OR = 1.37, 95% CI = 0.99–1.88, *P*=0.057), and distal metastasis (yes vs. no: OR = 1.13, 95% CI = 0.72–1.79, *P*=0.597), but SOX2 expression was correlated with gender (male vs. female: OR = 0.69, 95% CI = 0.5–0.94, *P*=0.02).

### 3.7. SOX2 Involves in Some Key Molecular Functions in CRC

**Figure 5:**
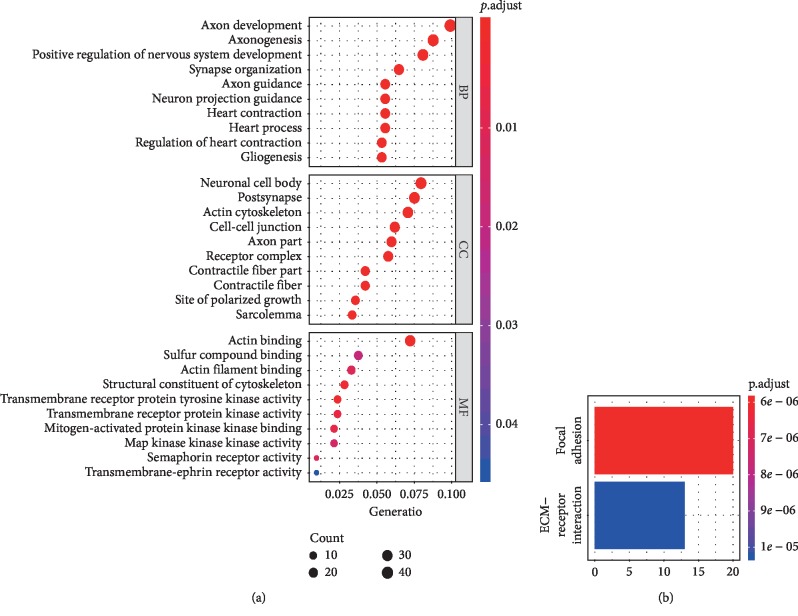
SOX2 involves in some cancer mechanisms, (a) GO (gene ontology) analysis; (b) KEGG (Kyoto Encyclopedia of Genes and Genomes) analysis.

GO and KEGG analyses showed that SOX2 involved in cell-cell junction, focal adhesion, extracellular matrix- (ECM-) receptor interaction, transmembrane receptor protein tyrosine kinase activity, transmembrane-ephrin receptor activity, semaphorin receptor activity, MAP kinase activity, mitogen-activated protein kinase binding, transmembrane receptor protein kinase activity, etc. in CRC (Figure 5), and these pathways are closely associated with cancer development.

### 3.8. Further Survival Analysis from Validation Data

**Figure 6:**
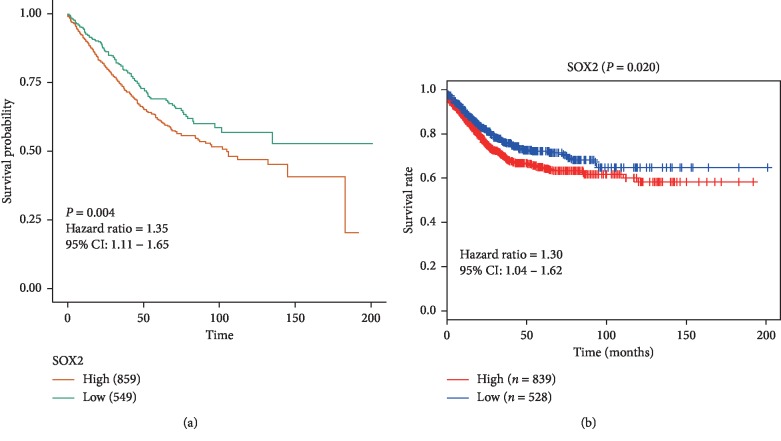
Survival analysis of SOX2 expression from validation data. (a) Overall survival (OS) in 1408 patients; (b) disease-free survival (DFS) in 1367 patients.

The validation data included 1408 patients with CRC, and the result also confirmed that SOX2 expression was significantly associated with worse OS (HR = 1.35, 95% CI: 1.11–1.65, *P*=0.004) (Figure 6(a)). Further validation data with 1367 CRC patients were used, and the result showed that SOX2 expression was associated with worse DFS (HR = 1.30, 95% CI: 1.04–1.62, *P*=0.02) (Figure 6(b)).

## 4. Discussion

CSCs contribute to tumor metastasis and prognosis and therapeutic resistance [45, 46]. CSCs may offer new promising therapeutic targets of treatment modalities applicable to human various cancers [47, 48]. SOX2 involves in the development and maintenance of stem-like properties in cancer cells [14, 15]. SOX2 involves in many signaling pathways such as VEGF, MAPK, Notch, P53, Wnt, and Jak-STAT, regulates many expression of genes, and regulates self-renewal and differentiation of stem cells, which may contribute to migration, invasion, and proliferation of cancer cells and the stemness of cancer stem cells, thereby affecting cancer progression, prognosis, and resistance toward anticancer therapies [14, 15, 17]. SOX2 expression is found across a wide range of human cancers such as breast cancer, lung cancer, and esophageal cancer [17]. SOX2 expression is correlated with the prognosis of some human cancers such as non-small cell lung cancer and cervical cancer with better prognosis [20, 21] and breast cancer and gastric cancer with worse prognosis [18, 19]. Studies have reported that SOX2 is frequently expression in CRC [6, 34, 35]. However, the function of SOX2 and its survival impact in patients with CRC are still largely uncertain. In the present work, we determined the clinicopathological effect of SOX2 and its expression on the prognostic impact of patients with CRC.

The relationships between SOX2 expression and the clinicopathological characteristics of CRC were investigated. The pooled data showed that SOX2 expression was not correlated with age and MSI status, which were consistent with the previous studies on age [23, 38] and MSI status [23, 24, 37, 39]. Further TCGA also showed no correlation with age and MSI status. Although no correlation was found between SOX2 expression and gender among all eligible studies, a large population with 441 cases reported that SOX2 expression was negatively correlated with gender and was lower in males than in females [23]. Further data from TCGA also demonstrated a negative association with gender, suggesting that more studies with larger sample sizes are needed to confirm this controversial finding. SOX2 expression was not associated with clinical stage, histological grade, tumor size, pT-stage, lymph node metastasis, and distal metastasis, which were in line with the previous publications regarding clinical stage [14, 35, 38], histological grade [14, 36, 41], tumor size [36], pT-stage [36, 38, 41], lymph node metastasis [14, 40], and distal metastasis [36]. Moreover, TCGA data also confirmed no significant association with clinical stage, lymph node metastasis, and distal metastasis. These results suggested that SOX2 expression was not significantly associated with progression and metastasis of CRC. Further relevant studies are necessary to confirm our results in the future.

The survival impact of SOX2 expression in patients with CRC was performed based on multivariate Cox analysis. SOX2 expression was not correlated with CSS but was significantly associated with worse OS in CRC. Additionally, further validation data from TCGA and GEO datasets (OS: *n* = 1408 CRC patients and DFS: 1367 CRC patients) demonstrated that SOX2 expression was significantly correlated with worse OS (HR = 1.35, *P*=0.004) and DFS (HR = 1.30, *P*=0.02). These analyses suggested that SOX2 was an independent prognostic marker for predicting poor prognosis. Additionally, the functional analysis showed that SOX2 involved in cell-cell junction, focal adhesion, extracellular matrix- (ECM-) receptor interaction, transmembrane receptor protein tyrosine kinase activity, transmembrane-ephrin receptor activity, semaphorin receptor activity, mitogen−activated protein kinase binding, transmembrane receptor protein kinase activity, MAP kinase activity, etc., which further suggested that SOX2 may be closely linked with CRC prognosis.

There are several limitations in the present study. First, this meta-analysis has not been registered online. Second, the main ethnic population was Asian and European; other ethnic groups, such as Africans, were lacking. Third, due to fewer studies, subgroups were insufficient and meta-regression analysis was not performed. However, heterogeneity analysis was carried out based on sensitivity analysis and the detailed reasons for the potential heterogeneity were not very certain. The possible use of the inappropriate and different conditions of IHC methods such as different/unclear cutoff values of SOX2 expression and different sources of anti-SOX2 antibody may lead to the potential sources of heterogeneity. For example, in the future, the expression of SOX2 is defined as positive or negative, which should be recommended using a uniform standard. Fourth, only Han et al. reported that SOX2 expression was correlated with DFS in 164 CRC cases (HR = 2.558, *P*=0.020) [6]. Finally, sample sizes on the analyses between SOX2 expression and the clinicopathological features and the prognosis were relatively small in the meta-analysis, more studies with large study population are necessary to further determine whether SOX2 is correlated with clinical and prognostic value in CRC based on IHC methods.

In conclusion, the current study suggested that SOX2 expression was not significantly correlated with age, gender, MSI status, clinical stage, histological grade, tumor size, pT-stage, lymph node metastasis, distal metastasis, and CSS but was associated with worse OS and DFS. Our data suggested that SOX2 may be used as an independent prognostic marker for worse prognosis in CRC. More large-scale prospective studies with larger sample sizes are required to further validate these findings.

## Figures and Tables

**Table 1:** Main characteristics of the eligible publications.

First author	Country	Age	Stage	Antibody	Sources of antibody	Staining	Cutoff values (IHC)	Cancer	Clinical features	Survival-MA
								Total (E+ %)		
Ong et al. [42]	Singapore	NA	1–4	Anti-SOX2	Clone 57CT23.3.4; Abcam, Cambridge, MA	Cytoplasm	10%	310 (NA)	No	CSS
Neumann et al. [41]	Germany	66.5	NA	Anti-SOX2	Clone D6D9, cell Signaling technology, Danvers, MA	Nuclei	10%	114 (21.1%)	Yes	NA
Han et al. [40]	China	NA	NA	Anti-SOX2	Epitomics	NA	NA	44 (20.5%)	Yes	NA
Lee et al. [39]	Korea	NA	1–4	Anti-SOX2	Abcam, Cambridge, UK	Nuclei	5%	110 (17.3%)	Yes	NA
Liu et al. [43]	China	NA	1–4	Anti-SOX2	Cell Signaling Technology, Inc	Nuclei	≥2 scores	67 (22.4%)	Yes	NA
Ma et al. [38]	China	65	1–4	Anti-SOX2	ab75485, Abcam, Cambridge, MA	NA	>1.40	89 (50.6%)	Yes	NA
Raghoebir et al. [37]	The Netherlands	NA	NA	Anti-SOX2	Immune systems	Nuclei	5%	135 (20.7%)	Yes	NA
Lundberg et al. [23]	Sweden	NA	1–4	Anti-SOX2	Abcam, Cambridge, UK	Nuclei	NA	441 (10.7%)	Yes	CSS
Yan et al. [36]	China	65	3–4	Anti-SOX2	Abcam	NA	2.5	280 (41.8%)	Yes	NA
Miller et al. [24]	Australia	66.7	3	Anti-SOX2	EPR3131; Abcam cell Signaling Technology, Danvers	Nuclei	NA	104 (18.3%)	Yes	CSS, OS
Zheng et al. [35]	China	NA	1–4	Anti-SOX2	Mass cat. no. 636675, EMD Millipore	NA	NA	47 (80.9%)	Yes	NA
Han et al. [44]	Korea	59.8	3	Anti-SOX2	Billerica, MA, USA	NA	≥1 score	164 (82.3%)	No	DFS
Li et al. [34]	China	NA	1–4	Anti-SOX2	#ab92494	NA	NA	432 (NA)	No	OS

NA: not applicable; IHC: immunohistochemistry; E+: positive/high expression; MA: multivariate Cox analysis; DFS: disease-free survival; OS: overall survival; CSS: cancer-specific survival.

**Table 2:** Summary of results from this meta-analysis.

Variables	OR [95% CI]	Heterogeneity (*p*)	*t* value	*P* value	Studies	Cases
Age (≥60 vs. < 60 years)	0.80 [0.13, 4.77]	0.618	−1.621	0.352	2	530
Gender (male vs. female)	0.91 [0.51, 1.64]	0.138	−0.439	0.684	5	991
MSI status (MSI vs. MSS)	1.40 [0.66, 3.00]	0.513	1.422	0.25	4	775
Clinical stage (stages 3-4 vs. 1-2)	1.34 [0.32, 5.64]	0.028	0.647	0.564	4	619
Histological grade (grades 3-4 vs. 1-2)	1.65 [0.62, 4.33]	0.004	1.428	0.227	5	999
Tumor size (≥5 vs. ≤5 cm)	0.78 [0.00, 1913.29]	0.018	−0.398	0.759	2	347
pT-stage (T 3-4 vs. 1-2)	1.12 [0.36, 3.43]	0.065	0.308	0.778	4	526
Lymph node metastasis (yes vs. no)	3.19 [0.58, 17.66]	0.368	2.916	0.1	3	225
Distal metastasis (yes vs. no)	1.26 [0.14, 10.91]	0.015	0.454	0.694	3	438

OR: odds ratio; CI: confidence interval; MSI: microsatellite instability; MSS: microsatellite stability.

**Table 3:** Correlation of SOX2 with the clinicopathological characteristics from the cancer genome atlas.

Variables	Comparison	OR with 95% CI	*P*	Cases
Age	≥60 vs. <60 years	1.03 (0.73–1.46)	0.859	618
Gender	Male vs. female	0.69 (0.5–0.94)	0.02	618
MSI status	MSI vs. MSS	1.08 (0.77–1.52)	0.663	618
Clinical stage	Stages 3-4 vs. 1-2	1.37 (0.99–1.89)	0.059	598
Venous invasion	Yes vs. no	1.12 (0.75–1.65)	0.585	536
Lymphatic invasion	Yes vs. no	1.27 (0.91–1.79)	0.162	557
Lymph node metastasis	Yes vs. no	1.37 (0.99–1.88)	0.057	615
Distal metastasis	Yes vs. no	1.13 (0.72–1.79)	0.597	545

OR: odds ratio; CI: confidence interval; MSI: microsatellite instability; MSS: microsatellite stability.

## Data Availability

Data are available in this manuscript.

## Supplementary Materials

Supplementary Materials Table S1: data of the eligible publications with the clinicopathological characteristics. Figure S1: publication bias using Egger's test. PRISMA 2009 Checklist Search terms Code for example.
