# Supplementary material for: Clinical and Survival Impact of Sex-Determining Region Y-Box 2 in Colorectal Cancer: An Integrated Analysis of the Immunohistochemical Study and Bioinformatics Analysis
Source: J Oncol. 2020 Feb 13;2020:3761535. doi: 10.1155/2020/3761535 (PMC7040407; doi:10.1155/2020/3761535)
Supplement: Supplementary Materials — Table S1: data of the eligible publications with the clinicopathological characteristics. Figure S1: publication bias using Egger's test. PRISMA 2009 Checklist Search terms Code for example. [file 3761535.f1.zip › Supplementary Materials/Search terms.docx]

Search terms

PubMed

(("colorectal neoplasms"[MeSH Terms] OR ("colorectal"[All Fields] AND "neoplasms"[All Fields]) OR "colorectal neoplasms"[All Fields] OR ("colorectal"[All Fields] AND "cancer"[All Fields]) OR "colorectal cancer"[All Fields]) OR ("colorectal tumour"[All Fields] OR "colorectal neoplasms"[MeSH Terms] OR ("colorectal"[All Fields] AND "neoplasms"[All Fields]) OR "colorectal neoplasms"[All Fields] OR ("colorectal"[All Fields] AND "tumor"[All Fields]) OR "colorectal tumor"[All Fields]) OR ("colorectal neoplasms"[MeSH Terms] OR ("colorectal"[All Fields] AND "neoplasms"[All Fields]) OR "colorectal neoplasms"[All Fields] OR ("colorectal"[All Fields] AND "carcinoma"[All Fields]) OR "colorectal carcinoma"[All Fields]) OR ("colorectal neoplasms"[MeSH Terms] OR ("colorectal"[All Fields] AND "neoplasms"[All Fields]) OR "colorectal neoplasms"[All Fields] OR ("colorectal"[All Fields] AND "neoplasm"[All Fields]) OR "colorectal neoplasm"[All Fields]) OR CRC[All Fields] OR ("rectal neoplasms"[MeSH Terms] OR ("rectal"[All Fields] AND "neoplasms"[All Fields]) OR "rectal neoplasms"[All Fields] OR ("rectal"[All Fields] AND "cancer"[All Fields]) OR "rectal cancer"[All Fields]) OR ("rectal neoplasms"[MeSH Terms] OR ("rectal"[All Fields] AND "neoplasms"[All Fields]) OR "rectal neoplasms"[All Fields] OR ("rectal"[All Fields] AND "tumor"[All Fields]) OR "rectal tumor"[All Fields]) OR ("rectal neoplasms"[MeSH Terms] OR ("rectal"[All Fields] AND "neoplasms"[All Fields]) OR "rectal neoplasms"[All Fields] OR ("rectal"[All Fields] AND "carcinoma"[All Fields]) OR "rectal carcinoma"[All Fields]) OR ("colonic neoplasms"[MeSH Terms] OR ("colonic"[All Fields] AND "neoplasms"[All Fields]) OR "colonic neoplasms"[All Fields] OR ("colon"[All Fields] AND "cancer"[All Fields]) OR "colon cancer"[All Fields]) OR ("colonic neoplasms"[MeSH Terms] OR ("colonic"[All Fields] AND "neoplasms"[All Fields]) OR "colonic neoplasms"[All Fields] OR ("colon"[All Fields] AND "tumor"[All Fields]) OR "colon tumor"[All Fields]) OR (("colon"[MeSH Terms] OR "colon"[All Fields]) AND ("carcinoma"[MeSH Terms] OR "carcinoma"[All Fields]))) AND (SOX2[All Fields] OR ("soxb1 transcription factors"[MeSH Terms] OR ("soxb1"[All Fields] AND "transcription"[All Fields] AND "factors"[All Fields]) OR "soxb1 transcription factors"[All Fields] OR "sox 2"[All Fields]) OR (Sex-determining[All Fields] AND ("geographic locations"[MeSH Terms] OR ("geographic"[All Fields] AND "locations"[All Fields]) OR "geographic locations"[All Fields] OR "region"[All Fields]) AND Y-box[All Fields] AND ("proteins"[MeSH Terms] OR "proteins"[All Fields] OR "protein"[All Fields]) AND 2[All Fields]) OR (("sex"[MeSH Terms] OR "sex"[All Fields]) AND determining[All Fields] AND ("geographic locations"[MeSH Terms] OR ("geographic"[All Fields] AND "locations"[All Fields]) OR "geographic locations"[All Fields] OR "region"[All Fields]) AND "Y"[Journal] AND box-2[All Fields]) OR (Sex-determining[All Fields] AND ("geographic locations"[MeSH Terms] OR ("geographic"[All Fields] AND "locations"[All Fields]) OR "geographic locations"[All Fields] OR "region"[All Fields]) AND Y-box[All Fields] AND 2[All Fields]) OR (SRY[All Fields] AND box-2[All Fields]))

Web of Science

主题: ((colorectal cancer OR colorectal tumor OR colorectal carcinoma OR colorectal neoplasm OR CRC OR rectal cancer OR rectal tumor OR rectal carcinoma OR colon cancer OR colon tumor OR colon carcinoma) AND (SOX2 OR SOX-2 OR Sex-determining region Y-box protein 2 OR Sex determining region Y box-2 OR Sex-determining region Y-box 2 OR SRY box-2))

EMBASE

('colorectal cancer'/exp OR 'colorectal cancer' OR (colorectal AND ('cancer'/exp OR cancer)) OR 'colorectal tumor'/exp OR 'colorectal tumor' OR (colorectal AND ('tumor'/exp OR tumor)) OR 'colorectal carcinoma'/exp OR 'colorectal carcinoma' OR (colorectal AND ('carcinoma'/exp OR carcinoma)) OR 'colorectal neoplasm'/exp OR 'colorectal neoplasm' OR (colorectal AND ('neoplasm'/exp OR neoplasm)) OR crc OR 'rectal cancer'/exp OR 'rectal cancer' OR (rectal AND ('cancer'/exp OR cancer)) OR 'rectal tumor'/exp OR 'rectal tumor' OR (rectal AND ('tumor'/exp OR tumor)) OR 'rectal carcinoma'/exp OR 'rectal carcinoma' OR (rectal AND ('carcinoma'/exp OR carcinoma)) OR 'colon cancer'/exp OR 'colon cancer' OR (('colon'/exp OR colon) AND ('cancer'/exp OR cancer)) OR 'colon tumor'/exp OR 'colon tumor' OR (('colon'/exp OR colon) AND ('tumor'/exp OR tumor)) OR 'colon carcinoma'/exp OR 'colon carcinoma' OR (('colon'/exp OR colon) AND ('carcinoma'/exp OR carcinoma))) AND (sox2 OR 'sox 2' OR 'sex-determining region y-box protein 2' OR ('sex determining' AND region AND 'y box' AND ('protein'/exp OR protein) AND 2) OR 'sex determining region y box-2' OR (('sex'/exp OR sex) AND determining AND region AND y AND 'box 2') OR 'sex-determining region y-box 2' OR ('sex determining' AND region AND 'y box' AND 2) OR 'sry box-2' OR (sry AND 'box 2'))
