# Supplementary material for: Clinical and Survival Impact of Sex-Determining Region Y-Box 2 in Colorectal Cancer: An Integrated Analysis of the Immunohistochemical Study and Bioinformatics Analysis
Source: J Oncol. 2020 Feb 13;2020:3761535. doi: 10.1155/2020/3761535 (PMC7040407; doi:10.1155/2020/3761535)
Supplement: Supplementary Materials — Table S1: data of the eligible publications with the clinicopathological characteristics. Figure S1: publication bias using Egger's test. PRISMA 2009 Checklist Search terms Code for example. [file 3761535.f1.zip › Supplementary Materials/Table S1.docx]

Table S1. Data of the eligible publications with the clinicopathological characteristics

| First author | Country | Sources of antibody | Staining | Cut-off values (IHC) | Cancer | ≥ 60 years | < 60 years | Male | Female | Stage 3-4 | Stage 1-2 | Grade 3-4 | Grade 1-2 | MSI | MSS | Tumor size ≥5 cm | Tumor size ≤ 5 cm | pT-stage 3-4 | pT-stage 1-2 | Lymph node metastasis (Yes) | Lymph node metastasis (No) | Distal metastasis (Yes) | Distal metastasis (No) |
| --- | --- | --- | --- | --- | --- | --- | --- | --- | --- | --- | --- | --- | --- | --- | --- | --- | --- | --- | --- | --- | --- | --- | --- |
|  |  |  |  |  | Total (E+ %) | E+/N | E+/N | E+/N | E+/N | E+/N | E+/N | E+/N | E+/N | E+/N | E+/N | E+/N | E+/N | E+/N | E+/N | E+/N | E+/N | E+/N | E+/N |
| Neumann 2011 | Germany | Clone D6D9,  Cell Signalling Technology,  Danvers, MA | Nuclei | 10% | 114 (21.1%) |  |  | 15/58 | 9/56 |  |  | 17/70 | 7/44 |  |  |  |  | 24/102 | 0/12 | 19/62 | 5/52 | 17/57 | 7/57 |
| Han 2012 | China | Epitomics | NA | NA | 44 (20.5%) |  |  |  |  |  |  |  |  |  |  |  |  | 9/25 | 0/19 | 8/26 | 1/18 | 7/21 | 2/23 |
| Lee 2013 | Korea | Abcam, Cambridge, UK | Nuclei | 5% | 110 (17.3%) |  |  |  |  |  |  |  |  | 3/12 | 16/98 |  |  |  |  |  |  |  |  |
| Liu 2013 | China | Cell Signaling Technology, Inc. | Nuclei | ≥2 scores | 67 (22.4%) |  |  | 11/43 | 4/24 | 7/31 | 8/36 | 6/19 | 9/48 |  |  | 5/42 | 10/25 |  |  | 4/14 | 11/53 |  |  |
| Ma 2014 | China | ab75485, Abcam, Cambridge, MA | NA | >1.40 | 89 (50.6%) | 28/56 | 17/33 | 31/59 | 14/30 | 3/11 | 37/65 |  |  |  |  |  |  | 39/73 | 6/15 |  |  |  |  |
| Raghoebir 2014 | The Netherlands | immune systems | Nuclei | 5% | 135 (20.7%) |  |  |  |  |  |  |  |  | 4/21 | 24/114 |  |  |  |  |  |  |  |  |
| Lundberg 2014 | Sweden | Abcam, Cambridge, UK | Nuclei | NA | 441 (10.7%) | 36/360 | 11/81 | 19/243 | 28/198 | 29/197 | 17/232 | 33/218 | 14/216 | 8/68 | 37/358 |  |  |  |  |  |  |  |  |
| Yan 2017 | China | Abcam | NA | 2.5 | 280 (41.8%) |  |  | 64/158 | 53/122 |  |  | 28/74 | 89/206 |  |  | 28/66 | 89/214 | 75/184 | 42/96 |  |  | 23/62 | 94/218 |
| Miller 2017 | Australia | EPR3131; Abcam | Nuclei | NA | 104 (18.3%) |  |  |  |  |  |  | 13/33 | 6/71 | 6/18 | 13/86 |  |  |  |  |  |  |  |  |
| Zheng 2017 | China | Cell Signaling Technology,  Danvers, Mass | NA | NA | 47 (80.9%) |  |  |  |  | 29/38 | 9/9 |  |  |  |  |  |  |  |  |  |  |  |  |

NA: not applicable; IHC: immunohistochemistry; E+: positive/high expression; N: the number of the study population; MSI: microsatellite instability; MSS: microsatellite stability.
