# Supplementary figures and images for: Clinical and Survival Impact of Sex-Determining Region Y-Box 2 in Colorectal Cancer: An Integrated Analysis of the Immunohistochemical Study and Bioinformatics Analysis
Source: J Oncol. 2020 Feb 13;2020:3761535. doi: 10.1155/2020/3761535 (PMC7040407; doi:10.1155/2020/3761535)

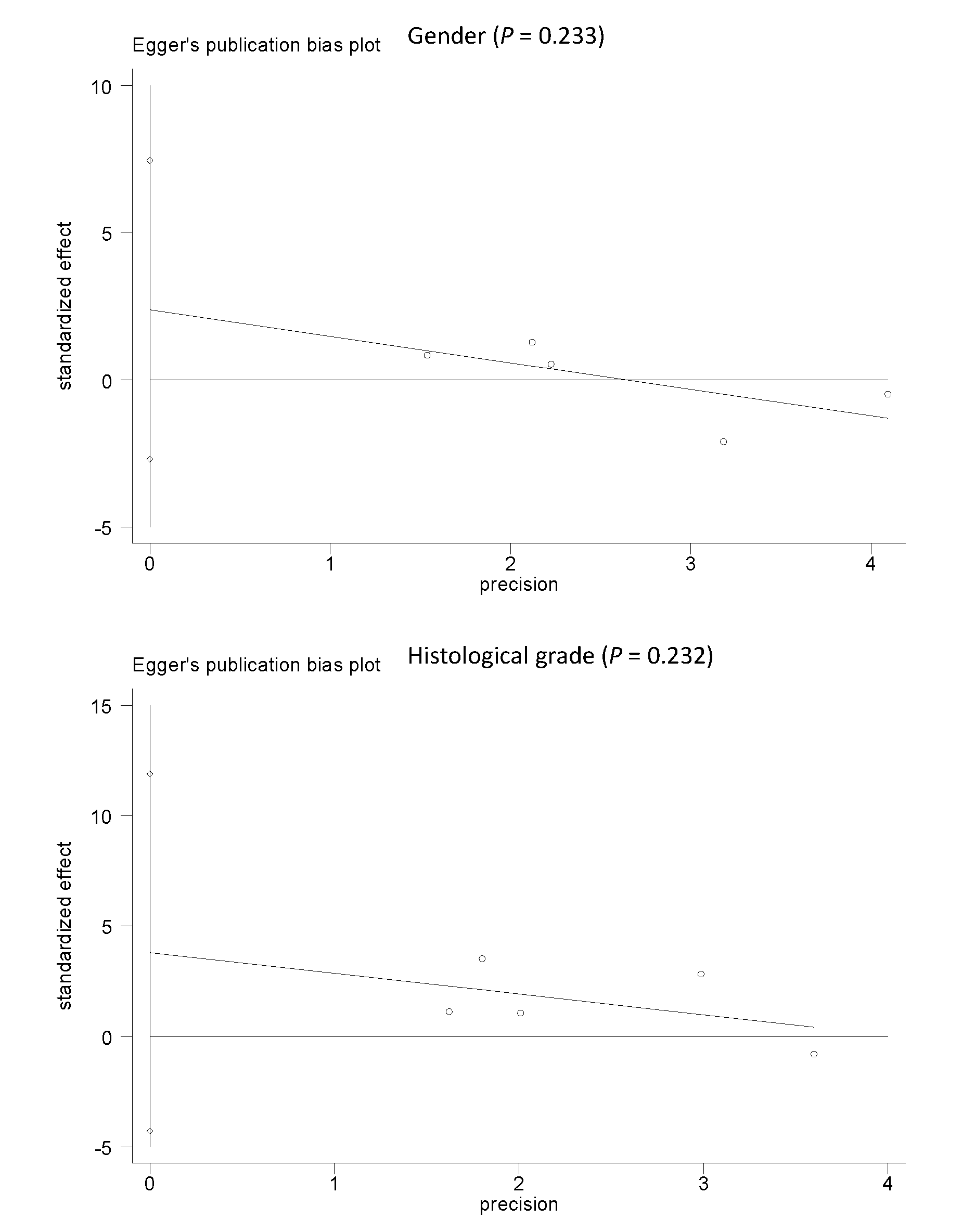

Supplement: Supplementary Materials — Table S1: data of the eligible publications with the clinicopathological characteristics. Figure S1: publication bias using Egger's test. PRISMA 2009 Checklist Search terms Code for example. [file 3761535.f1.zip › Supplementary Materials/Figure S1.tiff]
